# Lights and Shadows of Trait Emotional Intelligence: Its Mediating Role in the Relationship Between Negative Affect and State Anxiety in University Students

**DOI:** 10.3389/fpsyg.2020.615010

**Published:** 2021-01-15

**Authors:** Rocio Guil, Rocio Gómez-Molinero, Ana Merchán-Clavellino, Paloma Gil-Olarte

**Affiliations:** ^1^Department of Psychology, University of Cádiz, Cádiz, Spain; ^2^Institute of Social and Sustainable Development, University of Cádiz, Cádiz, Spain

**Keywords:** trait emotional intelligence, negative affect, state anxiety, mediation analyses, emotional attention, emotional clarity, mood repair, TMMS-24

## Abstract

Nowadays, students are experiencing difficult and stressful situations due to the Global Pandemic Alert. This changing world can evoke negative emotions that have been traditionally linked to higher anxiety. Researches have been focused on the positive outcomes of trait emotional intelligence (TEI) preventing psychological disorders. However, the possibility that TEI might have a dark side has been neglected. Hence, this study aimed to explore the mediating effect of the three dimensions of TEI in the relationship between negative affect and anxiety symptoms among college students. Participants of this research were 467 undergraduates who completed an online self-reported questionnaire including the Positive and Negative Affect Schedule (PANAS; Watson et al., 1988), the State-Trait Anxiety Inventory (STAI; Spielberger et al., 1970), and Trait Meta-Mood Scale (TMMS-24, Salovey et al., 1995). The global serial mediation model showed that the total amount of variance explained by the global model was 30.8% (*R*^2^ = 0.31). Negative affectivity and age accounted for the 15.1% of state anxiety variance (*R*^2^ = 0.15; c: *B* = 0.63, *p* < 0.001) while 15.7% of the variance of state anxiety was attributed to the direct or indirect effect of the three dimensions of TEI (*R*^2^ = 0.16). Five indirect effects presented statistical significance (95% BootCI). The contrast analyses between mediators showed that three indirect effects had higher statistical weigh; the ability of negative affect to increase state anxiety through (i) emotional attention; (ii) emotional clarity, and (iii) serially through emotional clarity and mood repair. Our results indicated that students’ negative emotions lead to higher emotional attention which in turn may enhance state anxiety in two ways: by a direct effect of emotional attention on state anxiety and by a serial effect through emotional clarity. Moreover, when negative affect is associated with lower emotional clarity, anxiety symptoms may also arise. However, when attention and clarity are connected, the negative effect is reversed into a positive one, decreasing state anxiety.

## Introduction

University life has been widely described as a period of change in which undergraduates have to adapt to academic pressures and different social and personal responsibilities. The higher education stage is also part of the transition to adulthood associated with frequent economic problems that may cause mental disorders (Rubley, 2017). According to the World Health Organization (World Health Organization [WHO], 2017), in 2017, 2,408,700 people were affected by anxiety disorders in Spain, highlighting university students as a risk group due to the psychological, social, and academic demands that they must face.

Moreover, university life has been also disturbed due to global pandemic COVID-19 (World Health Organization [WHO], 2020a). The COVID-19 has emerged as a public health crisis causing multiple changes and psychological problems or imbalances throughout the population (Wang et al., 2020; World Health Organization [WHO], 2020a,b). Specifically, in the university context, undergraduates have faced difficult and stressful situations in their academic climate such as virtual classes and exams, independent work, and isolation that have caused incomparable psychological distress, increasing negative emotional states (Mazza et al., 2020). In this regard, negative affect has been traditionally linked to lower academic performance, stress (Pollack and Herres, 2020), and symptoms of anxiety and depression (Toro et al., 2018).

Even though this global sanitary emergency is a recent problem, recent studies that evaluated the psychological status of university students have revealed that negative affect was associated with higher levels of anxiety (Cao et al., 2020). This higher prevalence of anxiety disorders has become a major problem for colleges and universities, who must develop programs to encourage students to properly regulate their negative emotional states during crises (Guil et al., 2019).

According to the State-Trait Anxiety Theory of Cattell and Scheier (1961), state anxiety can be defined as “feelings of tension and apprehension and heightened autonomic nervous system activity” (Spielberger, 1972, p. 145). Research has linked high levels of anxiety in university students to lower academic performance, poorer sleep quality and self-confidence (Lun et al., 2018), and lower quality of life (Pillay et al., 2016) and may contribute to alcohol and drug abuse, reduced empathy, and academic dishonesty (Ip et al., 2016).

In light of the risks and consequences of high levels of anxiety among university students, the literature has highlighted the role of trait emotional intelligence (TEI) as an adequate tool to face stressful situations and achieve emotional well-being, and successful academic performance in the university context (Wang et al., 2016; Parhiala et al., 2018). TEI is composed of three dimensions and can be defined as the ability to attend (emotional attention), understand (emotional clarity), and modify (mood repair) emotional states (Salovey et al., 1995). Many studies have confirmed the role of TEI as a predictor of positive emotional states and personal, social, academic, and work success (Brackett et al., 2011; Caruso et al., 2019; Guil et al., 2019). Research has also shown how emotions and affective states control our health and well-being (Vera-Villarroel et al., 2019; Sarrionandia and Mikolajczak, 2020).

However, some authors have been discussing that TEI may not always lead to positive outcomes. A recent meta-analysis has shown that higher TEI may have a detrimental effect on mental health in different contexts and call for research of the direct and indirect mechanisms that lead to the dark side of EI (Davis and Nichols, 2016). Other authors have linked higher TEI values to chronic stress (Ciarrochi et al., 2002), antisocial personality (Zhang et al., 2015), and even to smartphone cheating (Gentina et al., 2018).

Empirical research has also highlighted deleterious results in the psychosocial well-being of individuals regarding one TEI subscale: emotional attention. This dimension has been associated with rumination (Extremera and Fernández-Berrocal, 2005, 2006), with higher levels of symptoms in patients with anxiety and depression (Goldman et al., 1996; Salovey et al., 2002; Lizeretti et al., 2012), and with lower mental health (Extremera and Fernández-Berrocal, 2006). More recently, we also found works indicating that high levels of emotional attention can negatively influence well-being if it is not followed by adequate levels to understand and regulate emotions (Davis and Nichols, 2016; Cañero et al., 2019).

In undergraduates, emotional attention has been positively related to stress and negative affect and negatively to well-being and life satisfaction (Sánchez-Álvarez et al., 2015). Pena and Losada (2017) indicated in their mediation model that, in stressful situations, students with higher levels of emotional attention manage emotions less effectively and tend to perceive higher test anxiety. Another recent study has also shown that TEI mediates the relationship between self-esteem and state anxiety in a sample of university students, also confirming a negative association between self-esteem and emotional attention (Guil et al., 2019). The authors also indicated that the effect of self-esteem on emotional attention and its influence on clarity and emotional repair exerted a mediated influence with the power to reverse the positive link between self-esteem and anxiety (Guil et al., 2019). In this sense, the self-esteem turned from a protector to a risk factor enhancing anxiety levels. Hence, and based on previous research, we hypothesized that negative affectivity would increase emotional attention values and, in turn, state anxiety among university students, thus confirming the dark side of TEI.

Therefore, and given the increasing incidence and prevalence of anxiety disorders in university students caused by the health crisis of COVID-19 (Cao et al., 2020; Chi et al., 2020; Odriozola-González et al., 2020; Wang et al., 2020) and the scarce evidence exploring how and when TEI shows its dark side of TEI, this research examines the serial mediator role of the three dimensions of TEI in the relationship between negative affect and anxiety levels, after controlling for the influence of age.

## Materials and Methods

### Sample and Procedure

The sample was composed of 467 undergraduate Spanish students, 385 women (82.4%), and 82 men (17.56%) with a mean age of 21.79 years (*SD* = 5.19). All participants were enrolled in a Psychology Bachelor. Course distribution indicated that 57.6% of sample students were in the first course, 8.99% in the second course, 13.28% in the third course, 16.06% in the fourth, and finally 4.07% in the fifth course.

Participants completed an online self-reported questionnaire including the Spanish adaptation of the Positive and Negative Affect Schedule (PANAS; Watson et al., 1988), the State and Trait Anxiety Inventory (STAI; Spielberger et al., 1970), and Trait Meta-Mood Scale (TMMS-24, Salovey et al., 1995). The study was conducted according to the criteria set by the declaration of Helsinki and each subject signed an informed consent before participating in the study.

### Instruments

#### Negative Affect Schedule (Watson et al., 1988)

Affective states were measured through the Positive (PA) and Negative Affect (NA) Schedule (PANAS; Spanish adaptation by Sandín et al., 1999). This self-report instrument consists of two 10-item scales that assess feelings of enthusiasm and activation (positive affect) and aversive mood states such as disgust and nervousness (negative affect; Watson et al., 1988). Responses were collected on a 5-point Likert scale from 1 (very slightly or not at all) to 5 (extremely). This scale has sown adequate psychometric properties with a reliability coefficient of 0.87 for negative affect and 0.88 for positive affect. The present study only used the negative affect dimension showing and adequate reliability (α = 0.82).

#### State-Trait Anxiety Inventory (STAI; Spielberger et al., 1970)

The STAI is a two 40-item scale that assesses transient (state anxiety) and lasting levels of anxiety (state anxiety) [Spielberger et al., 1970; Spanish adaptation by Seisdedos (1982)]. For this study, only state anxiety was used to measure feelings of apprehension, tension, nervousness, and worry in the present moment. An example item was “I am tense; I am worried.” This test is answered based on a 1–4 scale to rate the intensity of the anxious feeling. Internal consistency coefficients for the scale have ranged from.86 to.95. In the present study, the state anxiety dimension showed excellent reliability (α = 0.91).

#### Trait Meta-Mood Scale (TMMS-24; Salovey et al., 1995)

The TTMS-24 is a self-reported questionnaire that measures self-perceptions of emotional abilities. It is composed of 24 items divided into 3 dimensions: emotional attention which evaluates the extent a person reflects upon their mood and emotions, emotional clarity which reveals how clearly people identify own and others emotions, and mood repair which identifies people’s ability to regulate emotional states (Salovey et al., 1995). Responses were given on a 5-point Likert scale ranging from 1 (strongly disagree) to 5 (strongly agree). The Spanish adaptation (Fernández-Berrocal et al., 2004) has shown adequate reliability and validity indexes for all 3 dimensions. In our sample, Cronbach’s alpha values were 0.88 for emotional attention, 0.90 for emotional clarity, and 0.87 for emotional repair.

### Statistical Analysis

Preliminary analyses including descriptive statistics and bivariate Pearson correlations were conducted for all study variables using SPSS 22 (IBM Corp. Armonk, NY, United States). The effect size of the relations between the research variables was interpreted attending to Pearson’s correlation coefficients: less than.29 small effect, between 0.30 and 0.50 medium effect, and above 0.50 strong effect size.

The serial mediation analysis was performed using Model 6 of PROCESS macro for SPSS. Negative affect was included as the independent variable, TEI dimensions as mediator ones, age as the covariable, and state anxiety as the independent variable. Following Preacher and Hayes (2004), the significance of the indirect effects was tested through bootstrap analysis. This technique calculates 95% confidence intervals (CI) resampling the study data 10,000 times. As a criterion of significance, if zero is not included in the 95% CI, the indirect effects are statistically significant.

Finally, to examine the presence of common method variance, Harman’s single-factor test was conducted (Podsakoff et al., 2003). Following this technique, we performed an explanatory factor analysis including all variables using unrotated principal components factor analysis (Podsakoff et al., 2003). Harman’s test assumes that if common method variance is presented in the data, one variable will account for more than 50% of the covariance in the independent and dependent variables (Podsakoff et al., 2003). Our results revealed that the general factor did not account for most of the variance (21.32%). Thus, we determined that no common method variance was present.

## Results

Preliminary analyses are included in Table 1. Mean variable values showed that both men and women showed adequate levels of EA, EC, and MR, and moderate values of negative affect and state anxiety. Pearson’s correlations indicated statistically significant but small correlations between negative affect and emotional attention (*r* = 0.27, *p* < 0.01) and emotional clarity (*r* = −0.11, *p* < 0.05), and moderate correlations with state anxiety (*r* = 0.39, *p* < 0.01). Age was also significantly related to emotional clarity (*r* = 0.13, *p* < 0.01). Sex did not correlate with any study variable.

**TABLE 1:** Descriptive statistics and Pearson bivariate correlations between the all study variables.

Variables	Women		Men		Total								
	*M*	*SD*	*M*	*SD*	*M*	*SD*	1	2	3	4	5	6	7
Sex							1	−	−	−	−	−	−
Age	21.72	5.36	22.13	4.34	21.79	5.19	0.30	1	−	−	−	−	−
NA	22.56	5.93	23.59	5.22	22.73	5.812	0.07	–0.02	1	−	−	−	−
EA	29.23	5.27	28.68	4.91	29.14	5.201	–0.04	–0.05	0.27**	1	−	−	−
EC	28.98	5.51	29.37	4.867	29.05	5.40	0.03	0.13**	−0.14*	0.13**	1	−	−
MR	29.19	5.83	29.61	4.69	29.26	5.64	0.03	0.07	0.07	0.01	0.31**	1	−
SA	16.06	9.43	16.33	9.37	16.10	9.41	0.01	0.01	0.39**	0.19**	−0.29**	−0.37**	1

NA, negative affect; EA, emotional attention; EC, emotional clarity; MR, mood repair; SA, state anxiety. *p < 0.05; **p < 0.01.

**FIGURE 1:**
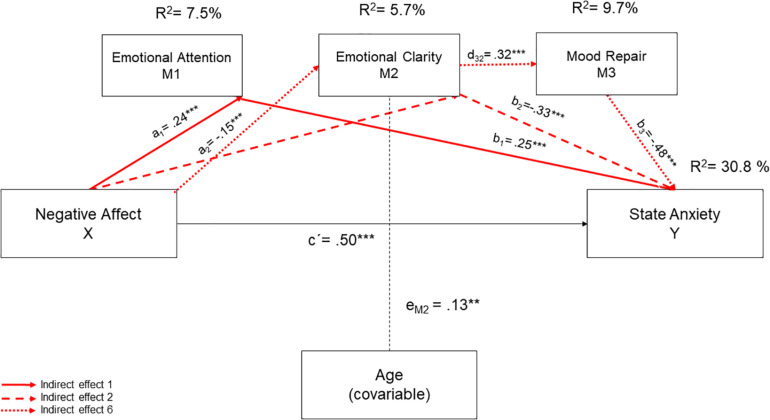
Protective effects for the serial mediation model proposed. To test the significance of the indirect effects, the bootstrapping technique was used to estimate the 95% confidence intervals. a, b, c’, d, and e = unstandardized regression coefficients. **p < 0.01; ***p < 0.001.

**FIGURE 2:**
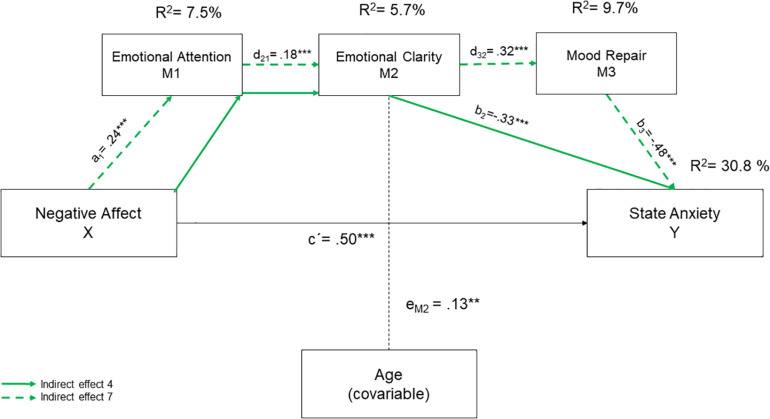
Risk effects for the serial mediation model proposed. To test the significance of the indirect effects the bootstrapping technique was used to estimate the 95% confidence intervals. a, b, c’, d, and e = unstandardized regression coefficients. **p < 0.01; ***p < 0.001.

Direct and indirect effects of the serial mediation model are shown in Figures 1, 2. The serial mediation analysis revealed that negative affect and age accounted for 15.1% of the unique variance in state anxiety (*R*^2^ = 0.15; *F* = 41.22, *p* < 0.001). Besides, 30.8% of variance was explained by the total model, which included negative affect, age, and the three dimensions of TEI (*R*^2^ = 0.31, *F* = 41.04, *p* < 0.01). Finally, 15.7% of the variance of state anxiety was credited to the direct or indirect effect of the three dimensions of TEI.

Considering the statistically significant direct effects, and as expected, negative affect was positive and statistically significant associated with state anxiety (*B* = 0.50; BootSE = 0.07; 95% BootCI = 0.37, 0.63), suggesting that high levels of negative affect lead to higher levels of state anxiety in undergraduate. Regarding the direct influence of TEI on state anxiety, we found different effects depending on the TEI dimension involved. Thus, EC (*B* = −0.33; BootSE = 0.07; 95% BootCI = −0.48, −0.19) and MR (B = −0.48; BootSE = 0.07; 95% BootCI = −0.62, −0.35) were negatively and statistically significant associated with state anxiety, indicating that high levels of EC and MR lead to lower levels of state anxiety, However, EA (*B* = 0.25; BootSE = 0.07; 95% BootCI = 0.10, 0.39) was positive and statistically significantly associated with state anxiety showing that high levels EA lead to higher state anxiety in our sample students.

Other direct effects showed that negative affect led to higher values of EA (*B* = 0.24; BootSE = 0.04; 95% BootCI = 0.16, 0.32) and lower levels of EC (*B* = −0.15; BootSE = 0.04; 95% BootCI = −0.23, −0.06). Moreover, EA was positively linked to EC (*B* = 0.18; BootSE = 0.05; 95% BootCI = 0.09, 0.29) and the latter with MR (*B* = 0.32; BootSE = 0.05; 95% BootCI = 0.22, 0.41). Age only affected EC in a statistically significant and positive way, indicating that older students perceive their emotional states more clearly.

According to the regression coefficient and considering that the CI (95%) did not include zero, we obtained five specific indirect effects. Two indirect effects contribute to decreasing anxiety levels (Figure 1), while three increased them (Figure 2). Concerning the two protective indirect effects, indirect effect 4 (a_1_d_2__1_b_2_) showed that higher levels of negative affect were associated with higher scores in emotional attention and emotional clarity, which in turn reduced state anxiety values (*B* = −0.01; BootSE = 0.01; 95% CI = −0.03, 0.01). Besides, indirect effect 7 (a_1_d_2__1_d_3__2_b_3_) showed that higher levels of negative affect decreased state anxiety values, by improving the three mediator values (B = −0.01; BootSE = 0.00; 95% BootCI = −0.01, −0.00).

Attending to the three risk indirect effects, indirect effect 1 (a_1_b_1_) revealed that higher levels of negative affect increased emotional attention levels and, consequently, anxiety levels (B = 0.06; BootSE = 0.02; 95% BootCI = 0.02, 0.11). Indirect effect 2 (a_2_b_2_) indicated that higher levels of negative affect increased trait anxiety through a decrease of EC (*B* = 0.05; BootSE = 0.02; IC 95% = 0.02, 0.09). Lastly, indirect effect 6 (a2d32b3) revealed that higher levels of negative affect increased trait anxiety values by acting in serially through a decrease in EC and MR (*B* = 0.02; BootSE = 0.01; 95% BootCIContrast) analyses between mediators were performed to determine which of the indirect effects had more statistical weight. The analyses showed that the three indirect effects that increase anxiety levels had the greatest statistical weight. Specifically, the capacity of negative affect to increase state anxiety through EA (Ind1), EC(Ind2), and serially through EC and MR (Ind6). These results indicated that when higher levels of negative affect are experienced, (i) more attention is paid to emotions and more anxiety is experienced; (ii) emotions are perceived less clearly and more anxiety is experienced; and (iii) students showed lower EC and MR which, in turn, increase state anxiety values.

## Discussion

Due to the scarce literature regarding the potential dark side of TEI, our study aimed to explore the effect that negative affect has on state anxiety among university students, as well as to examine the serial mediator role of TEI in the relationship between negative affect and anxiety levels. This research contributes to expand knowledge to the growing body of evidence highlighting that TEI may lead to maladaptive behaviors and negative health outcomes (Ciarrochi et al., 2002; Sánchez-Álvarez et al., 2015; Zhang et al., 2015; Pena and Losada, 2017; Guil et al., 2019).

Similarly to other studies, our sample, both men and women, showed adequate levels of EA, CE, MR, and moderate values of negative affect and state anxiety (Cazalla-Luna and Molero, 2014; Barraza-López et al., 2017). As stated, anxiety disorders are a common problem among university students and the COVID-19 pandemic has even increased the prevalence of negative affect and anxiety symptoms among undergraduates (Cao et al., 2020).

Preliminary analysis also confirmed previous research indicating that negative affect is linked to higher levels of state anxiety (Zvolensky et al., 2016; Toro et al., 2018), greater levels of EA, and lower EC (Palmer et al., 2002). Attending to the relationship between TEI and state anxiety, our findings revealed that this association was different depending on the TEI dimension involved. In this sense, EC and MR were negatively related to state anxiety among undergraduates. These results are in line with a vast pool of research that has verified the protective role of TEI on individuals’ health and well-being (Vera-Villarroel et al., 2019; Sarrionandia and Mikolajczak, 2020). However, our findings revealed that EA was positively correlated with anxiety symptoms suggesting that TEI may have a dark side. Hence, although the development of EC and MR constitutes a helpful tool to increase adaptation, higher levels of EA dimension can lead to non-beneficial and/or maladaptive behaviors (Goldman et al., 1996; Salovey et al., 2000; Extremera and Fernández-Berrocal, 2006; Lizeretti et al., 2012; Davis and Nichols, 2016; Serrano and Andreu, 2016; Pena and Losada, 2017; Cañero et al., 2019; Guil et al., 2019).

The results of the serial mediation model highlighted different influence paths between negative affect, TEI, and state anxiety. The direct effects confirmed that university students who presented higher levels of negative emotions showed higher levels of anxiety. Regarding the dimensions of TEI, the results confirmed again that EC and MR lead to lower levels of anxiety, while EA increased them, regardless of age. These findings provide scientific evidence to studies that indicate emotional clarity and mood repair act as protective factors promoting psychosocial adjustment and positive health outcomes (Extremera and Fernández-Berrocal, 2005). Moreover, our results also confirmed studies that concluded that high EA is not necessarily beneficial and excessive attention to one’s emotional states can cause negative psychiatric symptoms such as anxiety (Goldman et al., 1996; Salovey et al., 2000, 2002; Lizeretti et al., 2012). Therefore, higher levels of EA could act as a risk factor leading to ruminant thoughts that favor anxious states. Barraza-López et al. (2017) also indicated that higher levels of anxiety and depression can be linked to higher emotional attention levels and to reflect on these negative emotional states.

Even though it was not part of our main objective, our results also showed that age increased EC levels. This finding is consistent with the studies by Mankus et al. (2016), who indicated that the older the students, the better they understand their emotions. Besides, Rubenstein et al. (2015) also demonstrated in a longitudinal study that young people improved their levels of emotional understanding throughout adolescence. These results could reveal that age modifies TEI levels given that the different social, personal, and labor situations faced throughout life tend to improve the identification, labeling, and management of emotions. For this reason, it would be appropriate to propose intervention programs for the development of emotional competencies from an early age to compensate for the emotional deficits.

Finally, our mediation model also indicated that negative affect could influence state anxiety levels in university students by the indirect effect of TEI through 5 different pathways. Two indirect effects revealed that TEI might act as a protective factor, while three of them indicated that TEI could be a possible risk factor increasing state anxiety levels. Attending to the protective role of TEI, our results indicated that higher levels of negative affect were linked to higher scores in EA and EC, which in turn reduced the values of the state of anxiety. Along the same lines, we also found that higher levels of negative affect decreased state anxiety values, increasing all three mediator values. These results seem to be consistent with the literature that indicates that TEI benefits our health and acts as a protector factor fostering well-being (Vera-Villarroel et al., 2019; Sarrionandia and Mikolajczak, 2020). Concerning the dark side of TEI, we found that higher levels of negative affect increased emotional attention values and, in turn, anxiety levels. Besides, negative affect also reduced levels of EC, and this led to an increase in state anxiety. Finally, state anxiety was also increased in our undergraduates’ sample through the serial effect of negative affectivity diminishing EC and MR levels. The contrast analysis between mediators carried out confirmed that the three indirect effects that lead to an increase in anxiety had the greatest statistical weight. Particularly, our results indicated that negative affect increased state anxiety through EA and EC and serially through EC and MR. Although we have not found studies that analyze the mediating role of TEI between negative affect and state anxiety, our results seem to indicate that the apparent protective role of the TEI on the psychological adjustment of students can change, becoming a risk factor that promotes higher anxiety values. Moreover, these finds appear to confirm that TEI might have a dark side indicating that people with higher levels of EA manage emotions less effectively and tend to perceive greater anxiety (Goldman et al., 1996; Salovey et al., 2000; Pena and Losada, 2017; Guil et al., 2019).

Overall, the experience of negative emotions by students can lead to paying greater attention to their emotions, and this would increase state anxiety in two ways: by a direct effect of EA on state anxiety and by a serial effect through a decrease in EC. In addition, when negative affect lower EC values, symptoms of anxiety can also arise due to its direct effect on MR. However, when EA and EC are connected, the risk role of EA turns to a protective one decreasing state anxiety. Therefore, to evade the adverse effects of students’ negative affectivity on anxiety levels, intervention programs should focus on the parallel development of adequate levels of EA and EC to avoid the potential dark side of TEI.

This study has potential limitations that should be acknowledged. First, given the cross-sectional nature of this research, it is not possible to ascertain causal relationships between the measured variables. Besides, the specificity of the sample, composed of a fairly homogeneous group of undergraduates, makes it difficult to generalize our results to other populations. Second, the difference between women and men is considerable; however, we have not found correlations between sex and any study variables. In this sense, further studies are needed to explore the potential influence of sex in TEI, state anxiety, and negative affectivity. Finally, self-report instruments may lead to common method variance which can inflate or suppress the effects of relationships stated (Podsakoff and Organ, 1986). We conducted one-factor Harman’s test, and the results indicated that not a single factor accounted for 50% of the variance which indicated that our findings were not influenced by the common method variance.

Despite these limitations, this study highlights the need for administrations and educational institutions to include emotional intervention programs in the university training plan to enhance the academic and personal achievements of students and reduce psychiatry problems in undergraduates. These intervention programs will provide students with the necessary tools to adequately manage their negative emotional experiences to face new and challenging life experiences. Besides, these programs will help to reduce the high prevalence of anxiety disorders that appear during the university stage. Given the relatively scarce pool of literature, our study contributes to bringing light into the dark side of EI; however, future research should continue exploring the levels of EA that hinder personal and academic success and the way that EA is associated with the other TEI dimensions to avoid mental health problems.

## Data Availability Statement

The raw data supporting the conclusions of this article will be made available by the authors, without undue reservation, to any qualified researcher.

## Ethics Statement

The studies involving human participants were reviewed and approved by Ethics Committee of the University of Cádiz. The patients/participants provided their written informed consent to participate in this study.

## Author Contributions

RG, RG-M, and PG-O performed the data analysis and interpretation, wrote the manuscript, and approved the final version of the manuscript for submission. AM-C contributed to the data collection. All authors contributed to the article and approved the submitted version.

## Conflict of Interest

The authors declare that the research was conducted in the absence of any commercial or financial relationships that could be construed as a potential conflict of interest.
